# A giant plexiform schwannoma of the brachial plexus: case report

**DOI:** 10.1186/1749-7221-6-9

**Published:** 2011-11-01

**Authors:** Sho Kohyama, Yuki Hara, Yasumasa Nishiura, Tetsuya Hara, Tanefumi Nakagawa, Naoyuki Ochiai

**Affiliations:** 1 Dept. of Orthopaedic Surgery, University of Tsukuba, Graduate school of Comprehensive Human Sciences, 1-1-1 Tennodai, Tsukuba, Ibaraki 305-8575, Japan; 2 Showa University Hospital, 1-5-8, Hatanodai, Shinagawa, Tokyo 142-8666, Japan; 3 Senpo Tokyo Kowa Hospital, 3-10-11, Takanawa, Minato-ku, Tokyo 100-8066, Japan

**Keywords:** brachial plexus, giant, plexiform schwannoma

## Abstract

We report the case of a patient who noticed muscle weakness in his left arm 5 years earlier. On examination, a biloculate mass was observed in the left supraclavicular area, and Tinel's sign caused paresthesia in his left arm. Magnetic resonance imaging showed a continuous, multinodular, plexiform tumor from the left C5 to C7 nerve root along the course of the brachial plexus to the left brachia. Tumor excision was attempted. The median and musculocutaneous nerves were extremely enlarged by the tumor, which was approximately 40 cm in length, and showed no response to electric stimulation. We resected a part of the musculocutaneous nerve for biopsy and performed latissimus dorsi muscle transposition in order to repair elbow flexion. Morphologically, the tumor consisted of typical Antoni A areas, and immunohistochemistry revealed a Schwann cell origin of the tumor cells moreover, there was no sign of axon differentiation in the tumor. Therefore, the final diagnosis of plexiform Schwannoma was confirmed.

## Background

Plexiform Schwannoma is a rare variant of Schwannoma that accounts for only 5% of all Schwannomas, which typically shows a plexiform or multinodular growth pattern mimics plexiform neurofibroma. It was first described by Harkin and Reed in 1978 [1]. Plexiform Schwannoma usually develops in the dermis or subcutaneous tissue, and it is uncommon for the Schwannoma to develop in deep-seated nerves. Its histological features include Antoni A and B areas, diffuse and strong positivity with immunohistochemical markers like S-100, laminin and collagen type IV.

On the other hand, plexiform neurofibroma lacks Antoni A and B areas of schwannoma and shows weak S-100 positivity. Plexiform schwannoma shows up to 5% association with neurofibromatosis-2 and schwannomatosis, but has no association with neurofibromatosis-1 like plexiform neurofibroma [2,3]. This report describes the rare case of a giant plexiform Schwannoma in the brachial plexus.

### Case presentation

The patient was a 64-year-old male. More than 30 years earlier, he had experienced numbness in his left upper extremity. By his own account, he had undergone surgery and was diagnosed with a brachial plexus tumor. He stopped visiting the hospital after the surgery, no records of the surgery remain and the details thereof are unknown. The patient was diagnosed with diabetes mellitus 2 years earlier. He had no family history of a similar tumor. The patient noticed muscle weakness in his left arm 5 years earlier, but never visited a medical facility. Thereafter, the muscle weakness worsened, and he finally visited a local doctor 2 years earlier. His upper extremity was far from functional, and so he was referred to our hospital for further examination and treatment.

On examination a biloculate mass was observed in the left supraclavicular area, and Tinel's sign caused paraesthesia in his left arm. There was no evidence of the other tumor anywhere on his body. We found no café-au-lait spots or other signs of Recklinghausen disease neither.

Manual muscle testing of his left upper extremity revealed the following results: deltoid [1], biceps [1], brachioradialis [0], triceps [4], extensor digitorum [2], extensor digiti minimi [2], extensor pollicis brevis [2], extensor pollicis longus [2], extensor indicis [2], extensor carpi radialis [4], extensor carpi ulnaris [4], flexor carpi radialis [0], flexor carpi ulnaris [5], pronator teres [0], pronator quadratus [0], flexor digitorum superficiali [I-IV][0], flexor digitorum profindus (index finger) [0] and II-IV [5], flexor pollicis longus [0], flexor pollicis brevis [0], and flexor digiti minimi [0]. There was sensory loss in his axillary nerve, lateral brachial cutaneous nerve, and median nerve. The ulnar nerve was intact. Therefore, impairment of the lateral, posterior, and part of the median cord of the brachial plexus was suspected.

**Figure 1:**
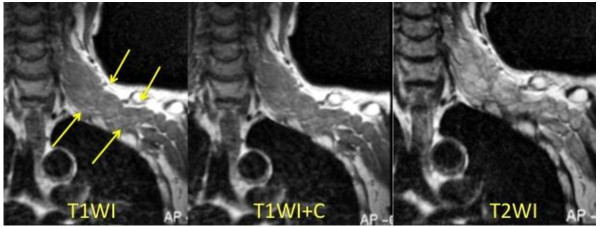
**MRI images**. Magnetic resonance imaging images. Magnetic resonance imaging showed a continuous, multinodular, plexiform tumor from the left C5 to C7 nerve root along the course of the brachial plexus to the left brachia. The tumor showed the same intensity as the muscle on T1-weighted images (T1WI), and slightly higher intensity on T2-weighted images (T2WI). Gadolinium-enhanced images (T1WI+C) showed no enhancement of the tumor. There was no sign of the tumor inside the vertebral canal.

There were no abnormal findings in his cervical radiography. Magnetic resonance imaging showed a continuous, multinodular, plexiform tumor from the left C5 to C7 nerve root along the course of the brachial plexus to the left brachia. The tumor showed the same intensity as the muscle on T1-weighted images and slightly higher intensity on T2-weighted images. Gadolinium-enhanced images showed no enhancement of the tumor. There was no sign of the tumor inside the vertebral canal (Figure 1).

**Figure 2:**
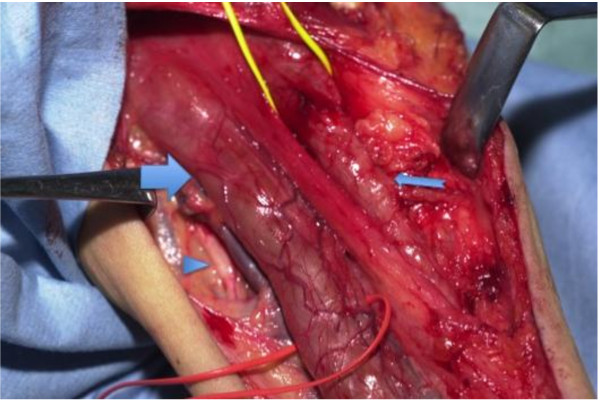
**operative findings**. Operative findings. The median and musculocutaneous nerves were extremely enlarged by the tumor and showed no response to electric stimulation. There was no abnormality in the ulnar nerve. Triangle; ulnar nerve Wide arrow; enlarged median nerve Narrow arrow; musculocutaneous nerve.

Tumor excision was attempted via exploration from the axilla to the cubital fossa. There was no abnormality in the ulnar nerve, but the median and musculocutaneous nerves were extremely enlarged by the tumor. Judging from the MRI and operative findings, the length of the tumor was approximately 40 cm in length(Figure 2). We haven't done electrophysiological examinations prior to the operation, so we performed electric stimulation during the operation. The tumor showed no response to it. Therefore, we terminated tumor enucleation, excised part of the musculocutaneous nerve (about 5 cm) for pathological examination, and performed latissimus dorsi muscle transposition in order to repair elbow flexion function. The latissimus dorsi muscle is innervated by C6-C8 nerve roots, mainly C7 nerve root. According to the patient's physical findings, C7 nerve root was possibly involved by the tumor. We have considered the transposition of the sternum part of the pectoralis major muscle which is innervated by C8-Th1 nerve roots, but since latissimus dorsi muscle maintained as strong muscle contraction as the pectoralis major, and we were confident of the latissimus dorsi muscle transposition from the past experiences, we have decided to perform the latissimus dorsi muscle transposition.

The excised specimen was formalin-fixed, paraffin-embedded, and sectioned for pathological evaluation. We performed hematoxylin and eosin staining, Masson trichrome staining, and immunohistochemistry for S-100 protein and neurofilamment.

**Figure 3:**
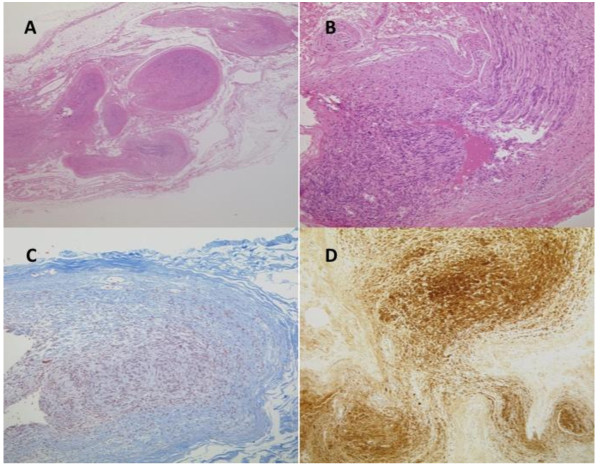
**Histological findings of the excised specimen**. **A. Hematoxylin and eosin staining (×40)**. Hematoxylin and eosin staining (×40). The tumor consisted of varying hypertrophic peripheral nerve fascicules showing a plexiform pattern, along with fibrous connective tissues.**B. Hematoxylin and eosin staining (×100)**. Hematoxylin and eosin staining (×100). Antoni A areas, which composed main part of the tumor. A nuclear palisading pattern was focally observed. **C. Masson trichrome staining (×100). **Masson trichrome staining (×100). The fibrous connective tissues around the tumor were roughly stained. **Immunohistochemistry for S-100 protein (×100). **Immunohistochemistry for S-100 protein (×100). The tumor cells were strongly positive for S-100 protein.

**Figure 4:**
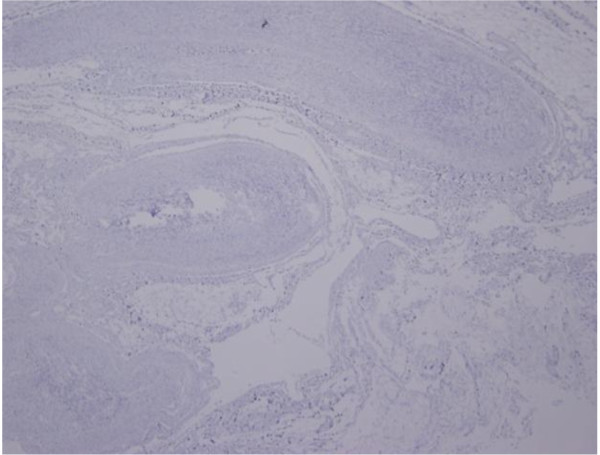
**Immunohistochemistry for neurofilament (×100)**. Immunohistochemistry for neurofilament (×100). There were no neurofilamment positive areas indicative of axons inside or outside the tumor.

Histologically, the tumor consisted of varying hypertrophic peripheral nerve fascicules showing a plexiform pattern, along with fibrous connective tissues (Figure 3-A). The tumor was mainly composed of Antoni A areas that showed dense fascicular and interlacing proliferation of spindle-shaped tumor cells without notable nuclear atypia. A nuclear palisading pattern was focally observed in the tumor (Figure 3-B). Typical Antoni B areas were not observed in the tumor. The fibrous connective tissues around the tumor were roughly stained with Masson trichrome (Figure 3-C). Immunohistochemistry results showed that the tumor cells were strongly positive for S-100 protein (Figure 3-D). There were no neurofilamment-positive areas indicative of axons located inside or outside the tumor (Figure 4).

Morphologically, the tumor consisted of typical Antoni A areas, which are generally found in Schwannomas but not in neurofibromas, and immunohistochemistry results revealed a Schwann cell origin of the tumor cells. These features made us suspect that the tumor was a Schwannoma. However, it is well known that Schwannoma-like lesions can be seen in some neurofibromas [4,5]. However in our case, there was absolutely no sign of axon differentiation in the tumor, and we observed no neurofilament-positive areas. Therefore, the final diagnosis of plexiform Schwannoma was confirmed.

**Figure 5:**
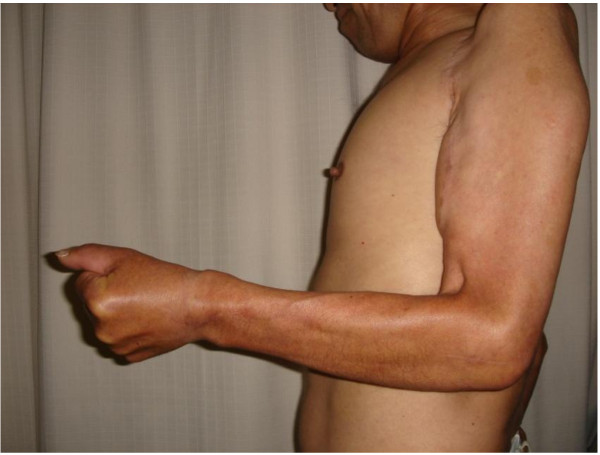
**Sixteen months after the operation**. Sixteen months after the operation. By latissimus dorsi transportation, the patient acquired elbow flexion up to 100 degrees.

Sixteen months after the operation, no progression in paralysis was observed. By latissimus dorsi transposition, the patient acquired elbow flexion up to 100 degrees (Figure 5). Collateral suture of the left flexor digitorum profundus I to flexor digitorum profundus II-IV, metacarpophalangeal joint arthrodesis of the left thumb, and reconstruction of the pollicis opponens with the Makin method improved his activities of daily living.

## Discussion

There have been few case report of plexiform Schwannomas in deep-seated nerves, as observed in our patient [6]. However, none of these reported Schwannomas was as large as that in our patient.

The differential diagnosis of plexiform Schwannoma with plexiform neurofibroma is very important. Schwannomas are benign encapsulated tumors originating from Schwann cells in the peripheral nervous system. Neurofibromas are benign, heterogeneous peripheral nerve sheath tumors arising from the connective tissue of peripheral nerve sheaths, especially the endoneurium. Neurofibromas are mostly solitary: however, when associated with neurofibromatosis type I, they can occur as multiple tumors. Optic glioma, Lisch nodules, and sphenoid dysplasia can also be found in neurofibromatosis type I, but non of them was found in our case. We must consider the possibility of neurofibromatosis type II. Definite criteria for neurofibromatosis type II include bilateral vestibular Schwannoma or first-degree family members of neurofibromatosis type II, and unilateral vestibular schwannoma diagnosed younger than 30 years or any two of meningioma, glioma, or schwannoma [7,8]. Another differential diagnosis is schwannomatosis. Schwannomatosis is generally a sporadic, nonfamilial condition that presents in older age groups with longer life expectancy, with two or more pathologically proven schwannomas. Definite criteria for schwannomatosis include the lack of neurofibromatosis type II mutations [5]. It is recently reported that trisomy of chromosomes 17 and 18 are present in a plexiform schwannoma [6]. We could not obtain approval of DNA analysis from the patient, but since his schwannoma was solitary, and there were no signs of vestibular schwannoma, glioma or meningioma, the possibility of neurofibromatosis type II and schwannomatosis were denied.

In our case, the clinical features and the appearance of the tumor was rather like that of plexiform neurofibroma. The patient did not show any café-au-lait spots, which precluded the presence of neurofibromatosis type I. The final diagnosis was thus based on microscopic tumor findings. In our case, the findings were specific to Schwannoma, namely, Antoni type A areas, anti-S-100 protein staining, minimal fibrous tissues, and no evidence of axon growth inside the tumor, which is most important. We excised only a part of the huge tumor; therefore, we cannot completely deny the possibility of Schwannoma-like lesions in a plexiform neurofibroma. However, since there were no neurofilament-positive areas inside or outside the tumor, we are confident in our diagnosis of Schwannoma.

In our case, the patient experienced paralysis for quite a long time, and we could thus not expect improvements in nerve function. In addition, because the tumor was too large to excise, we performed only a biopsy. Whether or not we should have instead resected the whole tumor is controversial. In past case reports of plexiform Schwannomas occurred in deep-seated peripheral nerves, paralysis was not present or very mild (Table 1). Furthermore, in all cases, the size of the tumor was much smaller than that observed in our case. Deep seated plexiform schwannoma can show necrosis and myxoid change in 12% of the lesions [6]. In our case, no such histological abnormality was seen in the excised specimen. However, considering the severe neurological deficit, necrotic change might have occurred somewhere in the tumor.

**Table 1:** Past case reports of plexiform Schwannoma in deep-seated peripheral nerves.

Reference	Case	Tumor site	Size	Paresthesia	Treatment
Oota et al	8 yr, M	C5,6 root	4 × 5 × 3 cm	None (postoperative +)	Enucleation

Ikeda et al	6 yr, F	Sciatic n	9 × 4 cm, 7 × 3 cm	None	Enucleation

Ikeda et al	14 yr, F	C4,5,6root	10 × 5 × 4 cm	None(postoperative +)	Enucleation, Reconstruction

Ikeda et al	14 yr, F	Median n.	1.5 × 2.5 cm	None	Enucleation

Nakamura et al	64 yr, F	L5 root	1.5 × 4 cm, 2 × 1 cm	None	Enucleation

Sakamoto et al	45 yr, M	Median n.		Atrophy of flexor carpi radialis, thenar eminence	Reconstruction

Maehara et al	42 yr, M	Sciatic n.	21 × 5 cm	None	Enucleation

Okuda et al	19 yr, F	Digital n.		None	Enucleation

Kawakami et al	28 yr, M	Median n.	3 × 3 cm	None	Enucleation

Oono et al	67 yr, F	Brachial plexus	3 × 4 × 5 cm	None	Enucleation

Horikiri et al	54 yr, F	Median n.	4 × 5 cm, 10 × 17 cm	Atrophy of thenar prominence	Enucleation

Kawamura et al	59 yr, F	Ulnar n.	35 cm	None	Enucleation, Reconstruction

We must consider the possibility of worsening paralysis and malignancy when contemplating tumor resection. If the tumor develops another fascicle, the paralysis may become worse. However, in our patient, considering his history the development of the tumor must have been very slow. Furthermore, according to the pathological diagnosis, the tumor was a plexiform Schwannoma, which is associated with a very low possibility of malignancy. Instead, we have found only one case report describing malignization of a plexiform Schwannoma [9].

## Conclusions

We have experienced the rare case of a giant plexiform Schwannoma in the brachial plexus. The affected nerves did not react to electric stimulation during the operation, and tumor invasion was extremely extensive, we therefore decided only to remove part of the tumor as biopsy, and changed the purpose of the operation from tumor removal the to reconstruction of elbow function. The diagnosis of plexiform Schwannoma had been made based on the pathological study. The condition of the patient remains satisfactory thus far, but careful observation of the progression of paralysis is necessary.

## Consent

Written informed consent was obtained from the patient for publication of this Case report and any accompanying images. A copy of the written consent is available for review by the Editor-in-Chief of this journal.

## Competing interests

The authors declare that they have no competing interests.

## Authors' contributions

SK participated in practice and operation, and constructed the manuscript. YH and YN participated in practice and operation, and helped to construct the manuscript. TH and TN carried out the operation. SS carried out pathological study. NO also carried out the operation, and have given final approval of the version to be published. All authors read and approved the final manuscript.
